# Biophysical Tools to Study Cellular Mechanotransduction

**DOI:** 10.3390/bioengineering4010012

**Published:** 2017-02-07

**Authors:** Ismaeel Muhamed, Farhan Chowdhury, Venkat Maruthamuthu

**Affiliations:** 1 Joint Department of Biomedical Engineering, University of North Carolina at Chapel Hill and North Carolina State University, Raleigh, NC 27695, USA; 2 Department of Mechanical Engineering and Energy Processes, Southern Illinois University Carbondale, Carbondale, IL 62901, USA; 3 Department of Mechanical and Aerospace Engineering, Old Dominion University, Norfolk, VA 23529, USA

**Keywords:** mechanotransduction, traction force microscopy, magnetic twisting cytometry, shear flow microfluidic device, integrins, focal adhesions, cadherins, adherens junction

## Abstract

The cell membrane is the interface that volumetrically isolates cellular components from the cell’s environment. Proteins embedded within and on the membrane have varied biological functions: reception of external biochemical signals, as membrane channels, amplification and regulation of chemical signals through secondary messenger molecules, controlled exocytosis, endocytosis, phagocytosis, organized recruitment and sequestration of cytosolic complex proteins, cell division processes, organization of the cytoskeleton and more. The membrane’s bioelectrical role is enabled by the physiologically controlled release and accumulation of electrochemical potential modulating molecules across the membrane through specialized ion channels (e.g., Na^+^, Ca^2+^, K^+^ channels). The membrane’s biomechanical functions include sensing external forces and/or the rigidity of the external environment through force transmission, specific conformational changes and/or signaling through mechanoreceptors (e.g., platelet endothelial cell adhesion molecule (PECAM), vascular endothelial (VE)-cadherin, epithelial (E)-cadherin, integrin) embedded in the membrane. Certain mechanical stimulations through specific receptor complexes induce electrical and/or chemical impulses in cells and propagate across cells and tissues. These biomechanical sensory and biochemical responses have profound implications in normal physiology and disease. Here, we discuss the tools that facilitate the understanding of mechanosensitive adhesion receptors. This article is structured to provide a broad biochemical and mechanobiology background to introduce a freshman mechano-biologist to the field of mechanotransduction, with deeper study enabled by many of the references cited herein.

## 1. Mechanotransduction

Mechanotransduction is an umbrella term to describe any biochemical cellular response generated against specific extracellular or intracellular mechanical stimuli [1,2,3,4,5]. More precisely, mechanotransduction is the process that converts mechanical force into specific biochemical signals [6,7]. The process involves force reception (mechanosensitivity/force recognition), force transduction and a corresponding response [8,9]. The biomechanical activation of certain membrane, cytosolic and nuclear proteins (e.g., growth factor associated adhesion receptors, cytoskeletal filaments, kinases and phosphatases, lamins, linker of nucleoskeleton and cytoskeleton (LINC) complexes and histone modifying proteins) that sense and/or transduce force is well established [4,10,11]. A whole host of elements are involved in the consequent cellular responses including, but not limited to, membrane associated protein classes such as stretch-activated ion channels, cell-cell adhesion receptors (cadherins, ICAM), gap junctions, cell-matrix adhesion proteins (integrins, syndecans, CD44), extracellular matrix proteins (fibronectin, collagen isoforms, laminin isoforms, proteoglycans, basement membrane), cytoskeletal components (actin microfilament, microtubule and intermediate filaments), nuclear lamins and associated proteins, growth factor receptors (epidermal growth factor receptor (EGFR), vascular endothelial growth factor receptor (VEGFR), platelet derived growth factor receptor (PDGFR)), and intracellular tension regulating components (myosin motors) [5]. These elements under specific mechanosensory stimulation regulate physiological functions [3,12]. 

### 1.1. Mechanosensitivity

**Figure 1:**
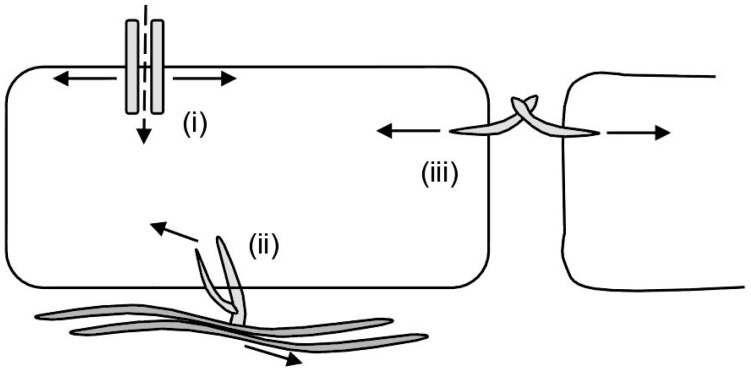
Three representative modes of mechanosensing by cells. Cells can use (**i**) mechanosensitive ion channels and receptors; (**ii**) mechanosensitive cell-ECM (extracellular matrix) interactions; and (**iii**) mechanosensitive cell-cell interactions. Solid arrows indicate forces acting on the mechanosensitive elements and the dashed arrow indicates ion conduction.

Cells sense their environment using specific cell surface receptors, and some are classified as mechanosensors when they sense mechanical cues and induce appropriate biochemical events (consequently the term mechanoreceptors) [2,12,13]. The ability to recognize specific mechanical stimuli is mechanosensitivity [14,15,16] (Figure 1). Mechanosensing receptors induce a variety of biochemical signals like conformation changes within the protein [17,18,19,20,21], biochemical activation and/or the recruitment of certain molecules [18,20,22,23,24]. These mechanically induced events modulate cell functions like migration [25], cell polarity [26,27], proliferation [28], differentiation [1], apoptosis [29,30], gene expression [8,31], and enable cells to adapt to the mechanical stimulus. Defects in mechanotransduction pathways are implicated in diseases such as muscular dystrophies [29], cardiomyopathies, atherosclerosis [13] and cancer metastasis [32]. 

### 1.2. Mechanotransduction Related Diseases

Mechanotransduction related cell signals have a critical role in the maintenance of tissues (muscle, bone, cartilage and vessels [29]), directing stem cell fate [1,33] and cell morphogenesis [34,35,36]. A common feature among many mechanotransduction related diseases is the disruption in force transmission between the extracellular matrix (ECM), cytoskeleton and/or nucleoskeleton [29], which either delay or affect the signal threshold in producing the necessary conformation change or activation of the mechanosensory complex. 

An example of a well-known mechanotransduction phenomenon is how inner ear hair cells transduce mechanical vibratory force into neural signals. The human ear has 3 major parts—outer, middle and inner ear. The outer ear receives and directs sound waves to the middle ear through the eardrum, the middle ear has many tiny bones that connect sound waves to the inner ear and the inner ear houses the cochlea, which is lined by inner ear hair cells in the organ of Corti [37,38]. These cells have fine hair like bundle protrusions called stereocilia that are arranged hexagonally [37,39]. The stereocilia are aligned in the order of their heights and are connected at their tips through tip link proteins. Mechanical vibratory forces cause displacements (tip elongation) in the serially height-wise organized tips of hair cells [39]. The sound waves deflect the arranged hair cells and produce tension in the connected tip links between the organized sterocilia [38]. This tension opens up mechanically gated ion channels and/or extend the elastic ankyrin protein repeats [39]. Any defect in tip link extension or mutations in cadherin 23 functional modules cause a loss in hearing [39]. 

Other examples of mechanically sensitive ion channels include TWIK related K^+^ channel (TREK), TWIK-related arachidonic acid stimulated K^+^ channel (TRAAK) and piezo (Fam38A and Fam38B) channels. TREK and TRAAK are mechanosensitive K^+^ channels which are mechanically activated form the lipid membrane in the absence of other cytosolic components [40,41]. Piezo channels are expressed in many organs including kidney, bladder, and lung, and on stretching rapidly allow ion passage [42]. The piezo protein homo-associates, forming a pore that is selective for cation transport [43,44], and in vitro purified piezo proteins embedded within lipid bilayers are mechanically sensitive to membrane perturbation, suggesting independence from other proteins in inducing mechanosensitivity [45]. Piezo co-immunoprecipitates with polycystin (a member of the transient receptor potential (TRP) family), stomatin-like protein 3 (STOML3) and trefoil factor family1 (TFF1) in different cell lines [46]. Polycystin inhibits [47], while interaction with STOML3 amplifies piezo mechanosensitivity [48]. The mechanical stretching and activation of piezo increases cytosolic Ca^2+^ and release of ATP in urothelial cells [49], and several human syndromes are associated with piezo mutations. Some include Dehydrated Hereditary Stomatocytosis (DHS), Distal Arthrogyposis Type 5 (DA5), Gordon Syndrome and Marden-Walker Syndrome (MWS). In fact, piezo knockout in mice is embryonically lethal [46]. 

Another example of a human disease arising from loss of mechanotransduction signals is atherosclerosis. Laminar flow of blood across the apical layer of endothelial cells exert an atheroprotective effect on cells, compared with inflammatory responses triggered by turbulent flow within the vessel [13,29,50]. Endothelial cells in contact with blood align in the direction of shear stresses, while the orthogonal stress produces strain normal to vessel walls and stretches the vessel diameter [13]. Disturbances in blood shear (turbulent flow) cause atherosclerotic plaques in vessel walls, which narrow arteries and contribute to vascular diseases and heart failure. Altering blood flow or the inability to sense shear can affect the embryonic development of the heart [51] and cause inflammation and increased plaques in adults with cardiovascular disease risk factors (diabetes, obesity, lack of exercise and smoking) [13]. 

Skeletal and cardiac cells also respond to external mechanical load following Wolff’s principle (sustained increase in the magnitude of applied stress on the bone enhances bone growth and remodeling [52]). In bone matrices themselves, induced pressure gradients (gravity and muscle induced contractive forces) produce deformations within the bone that drive interstitial fluids through the lacunae-canalicular network. This fluid flow induces bone remodeling and maintenance [53]. Osteoporosis and loss of bone mass result from altered fluid shear, abnormal nano-mechanical stress reception and response [54].

## 2. Tools to Study Cellular Mechanotransduction

**Figure 2:**
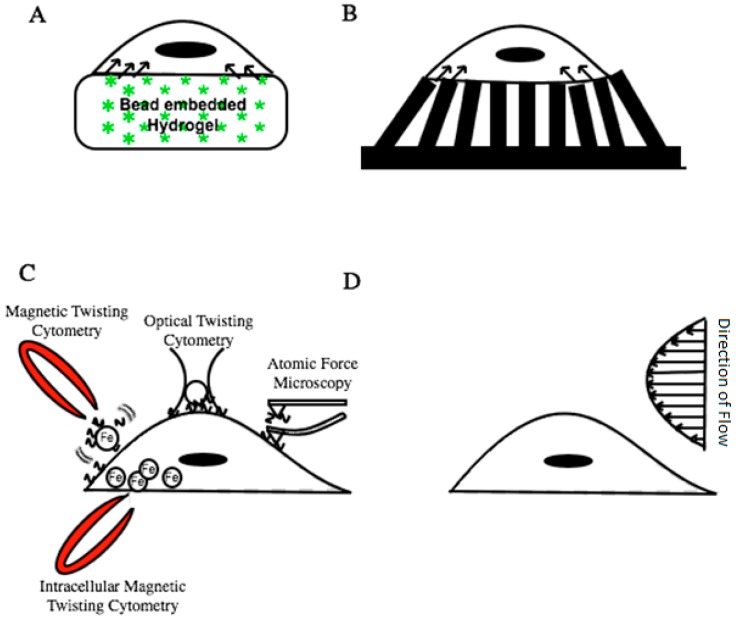
Common biophysical tools used to investigate mechanotransduction. (**A**,**B**) The Traction Force Microscopy set up quantifies the displacement in the substrate caused by the cell. Cells are grown on protein-functionalized (**A**) hydrogels embedded with fluorescent markers; or (**B**) micropillars with known height and elastic moduli. The deflection (Δl of a region of the hydrogel or pillar) is indirectly used to quantify cell contractility in that zonal area; (**C**) Magnetic or optical twisting cytometry and atomic force microscopy are techniques that can apply pico to micro Newton shear, compressive and tensile forces on cell receptors, and cytosolic organelles; (**D**) Shear forces from fluid flow are investigated by growing cells on tubular or cylindrical surfaces. The apical surface of the cell experiences shear forces that is proportional to flow velocity shear rate and viscosity.

To investigate the biophysical parameters that control cell mechanotransduction, engineers and biologists have developed different physical and molecular probes that quantitatively apply and/or detect pico-Newton forces (Figure 2).

Current bioengineered devices can generate forces that mimic inter- and intra-cellular forces produced across cell receptors. ECM properties (rheology, anisotropy, ligand density and epitope availability) determine cell deformation and stress generation [55]. Using probes customized with receptor ligands, the mechanosensing ability and force specificity of receptor/ligand pairs can be investigated. Depending on the directionality and type, forces can be classified into shear, tensile, compressive or oscillatory (rotatory) forces, and have been shown to induce direction specific biochemical signaling [56]. Almost all the developed force application tools are compatible with fluorescence or bright field microscope systems, enabling live cell investigation (across spatial or temporal dimensions). This report will briefly discuss the tools and forces applied in 2D (*x*-*y* plane) and the reader is advised to read other excellent articles discussing mechanotransduction effects of 3D forces [57,58,59,60,61,62]. 

### 2.1. Traction Force Microscopy (TFM)

TFM (Traction Force Microscopy) quantitatively measures the stress exerted by a cell on its substrate environment. Each cell is continually experiencing an internal force balance between compressive and tensile elements (the tensegrity model) and is influenced by other elements in the cellular environment [10,12,63,64]. The force exerted by cells on the environment is indirectly reflected on the intracellular force balance (prestress) and serves as an indicator of cell contractility. The preexisting tensional stress on an element/node before any external stress is applied is defined as prestress. The features of prestress (extent of rigidity, orientation and force balance of tensile and compressive elements) determine the extent of resistance in enduring displacement (strain), while attaining new stress equilibrium configurations [63]. The material properties of the cell can also, in turn, dictate the extent of spreading and differentiation in embryonic stem cells [65]. 

Adherent cells exert (traction) forces on their substrate by anchoring onto specific ECM proteins. The biochemical mechanism of traction generation involves a complex interplay between adhesion molecules, cytoskeletal elements, motor proteins, enzyme activity and their respective activation or recruitment kinetics [34,66,67]. The generated cell traction is measured by averaging the magnitude of forces that induce a substrate displacement per unit area [68]. By having fiducial fluorescent markers within a flexible substrate under the cell, and tracking its position with and without the cell, the substrate displacement is quantified. There are many custom programs to calculate 2D bead displacement (particle image velocimetry (PIV), particle tracking—e.g., Utrack 2.0, a matlab based program from the Danuser lab, UT Southwestern). From the displacement and the elastic modulus of the gel, the traction stress is calculated using analytical solutions (such as the Boussinesq solution) in Fourier space as explained by Butler et al. [68] or using the Finite Element Method (FEM) [69,70]. Here, the measured traction is the root mean square of the local forces per unit area and is calculated using Fourier Transform Traction Cytometry (FTTC) as outlined above. The displacement in the *z* direction is either negligible or ignored in 2D TFM, while the displacement in the *x* and *y* plane is often considered in a semi-infinite medium substrate (valid when lateral cell dimensions and displacements are smaller compared to gel thickness) [68]. Using hydrogels as substrates, the traction stresses (*σ*) are related to displacements by a tensorial version of Hooke’s law [71] with the known Young’s modulus (*E*) and Poisson’s ratio (*υ*) of the gel. For experimental purposes, the chosen Poisson ratio is 0.48–0.5 for polyacrylamide [72,73] and 0.5 for polydimethylsiloxane (PDMS) [74] gels.

Traction forces are generally determined for single cells or cluster of cells [75,76] and the required displacement map is calculated taking stage drift into account [62,68,77,78]. The major disadvantage with the setup is that high throughput data collection requires fine microscope mechanical stage *x*-*y* displacement with multi-dimensional data acquisition software with minimal change in the focal plane while acquiring multiple cell traction data points. Since cells are dynamic and frequently remodel (change in the contact area, adhesion strength under different cell cycle stages) it is sometimes difficult to compare traction force distributions at different time points. In a recent work, the need for removing attached cells to calculate substrate traction is avoided using well-arranged reference quantum dots onto a monocrystalline array substrate [79]. Another method to measure substrate deformations is by using a cholesteryl ester coated liquid crystal (LC) as a substrate for cells that can detect and transduce cellular traction forces [80].

Apart from using hydrogels, 2D traction forces can also be measured by growing cells on ECM functionalized micropatterned pillar substrates [76]. Each pillar can be used as a force transducing cantilever beam, by adhering cells onto ECM functionalized pillar surfaces. Cells exert traction stresses on the micropillar and cause the pillar to bend, which is then quantified. By altering the pillar height and material characteristics one can tune the modulus of rigidity of the substrate. For pillar-based studies (with known Young’s modulus (*E*), height (*L*), diameter (*D*)) on fixed substrates, the lateral force (in the linear regime of pillar deformation *δ*) at the top of pillar is given by [81,82],

*F* = K_bend_. *δ* = (3·π·*E*·*D*^4^/64·*L*^3^)·*δ*

By measuring the pillar deflection (using custom tracking software), the force generated by the cell to bend the pillar is quantified. The micropillar traction technique involves discrete displacements of individual adhesion zones, while gel-based TFM involves continuous substrates. The inconvenience of the micropillar data analysis is that the cell is grown on a layer of cylindrical needle like micropillars with controlled spacing that limits the maximum focal adhesion size and the number of focal adhesions and therefore may not represent physiological substrate adhesion in these respects. The major advantage is that high throughput data acquisition and time chase experiments are simpler as initial un-deflected pillar positions are known and cells do not need to be detached from substrates to quantify traction forces. 

While Traction Force Microscopy itself identifies absolute cell stresses and stress heat maps for single cells or cell clusters, TFM in combination with cell imaging has been used to track and correlate changes in cytoskeletal and associated signals with cell mobility [83,84], directionality of migration [58,60,82,85], heterogeneity of cell traction associated with proliferation [82,86] and stem cell fate [31,87,88]. 

### 2.2. Magnetic Twisting/Pulling Cytometry (MTC/MPC)

Magnetic twisting cytometry (MTC) is a well-established method to study the mechanical properties of living cells by applying a quantified external stress on a cell and measuring appropriate displacement parameters [12,65,89,90,91,92,93]. The 2D MTC setup consists of a horizontal and vertical Helmholtz coil around a mechanical stage [10,12]. The current flowing through the coil is controlled using an electronic controller, which modulates the magnetic field. Specific protein or peptide coated ferromagnetic microbeads are attached to the apical surface of the cell. The beads are magnetized by applying a strong and short magnetic pulse (~1000 G for less than 0.5 milliseconds). Following magnetization, a weak twisting field (much lower than the initial magnetizing field, ensures the beads will not be re-magnetized) is applied in the orthogonal direction using a vertical Helmholtz coil to the magnetic moment of the bead. As a result, the bead will attempt to align in the direction of the applied field, and due to partial embedment of the bead on the cell surface, the rotation of the bead is restricted. Therefore, such induced rotation will exert an out-of-plane shear stress. The degree of rotation is dependent on the cellular properties. It is to be noted that MTC induced shear stress can stimulate localized deformations within the cytoskeleton without disturbing the whole cell. On soft cells with some degree of bead embedment, there will be a larger bead displacement, while on stiffer cells, the resulting displacement will be small. By tracking bead displacement while applying external stress to the cell, MTC allows the measurement of cellular mechanical properties in live cells in real time. By regulating the amplitude and frequency of current passing through the coils, one can control the amount of shear stress on the cell surface. The magnetic field-induced bead displacement is tracked using a bead tracking software from which the complex modulus (G*) is computed. Measuring the phase lag between the input current in the coil and the resultant displacement allow the determination of G’ (elastic modulus) and G’’ (viscous modulus) of the cell. 

Magnetic pulling cytometry (MPC) works on a similar principle as MTC, except that a superparamagnetic bead is used. As the name suggests, the beads are pulled using a magnetic field and the tension induced bio-mechanochemical responses on cell receptors is investigated [94,95,96,97]. Using a permanent or electromagnetically controlled magnetic needle positioned close to a cell bound superparamagnetic bead, the bead can be magnetized [98,99]. The distance between the magnetic needle and the superparamagnetic bead determines the force applied and needs to be calibrated with the same bead-needle distance in experimental conditions. The pulling force applied through the magnetic needle is often calibrated by applying force on a bead embedded in a viscous fluid and by using the Stokes equation for low Reynolds number flow (force = 3π*ηDυ*, where *η* is viscosity, *D* is bead diameter, *υ* is bead velocity) [94]. The bead displacement is tracked optically. The magnetic pulling force can be applied for longer durations from minutes to days (to investigate longer period cellular morphological changes), supplemented with a broader magnetic field gradient by altering the magnetic strength or distance between needle and bead [98].

To specifically attach the magnetic bead to specific cell receptors, a magnetic bead with a surface exposed carboxyl or thiol group is covalently coupled to receptor ligands using specific tagging chemistry (for example free amines in protein ligands are linked to carboxyl beads using 1-Ethyl-3-[3-dimethylaminopropyl]carbodiimide (EDC), *N*-hydroxysuccinimide (NHS) chemistry) [12,18,100]. The major advantage of the MTC and MPC setup is that it can be optimized for high throughput data collection. The inconvenience is that the setup requires finely calibrated and magnetic field control equipment. Since commercially obtained magnetic beads can be of non-uniform sizes, the ligand functionalization chemistry needs to be optimized for uniform density coverage. 

### 2.3. Optical Tweezers

Originally developed for trapping atoms and molecules [101,102], optical tweezers have emerged as one of the important tools in biophysics research and have facilitated mechanistic single molecule studies in biology. Some of the seminal works carried out with optical tweezers with single molecule perturbation and resolution involves myosins [103,104,105], kinesins [105,106,107], dyneins [108,109,110], bacterial flagellar motors [111,112], DNA and RNA mechanics and related protein interactions [113,114,115,116,117,118,119,120]. Optical tweezers have also been widely used in cell studies [121,122,123] and in calibrating single molecule force sensors for cellular force spectroscopy [124,125,126]. 

The working principle of optical tweezers relies on the force induced by a laser beam focused onto small dielectric beads. The slimmest region of the focused beam encloses the strongest electric field gradient and traps the dielectric particles. Exploiting the law of conservation of momentum, the highly focused beam cause the bead to experience a force in the direction of the beam. This force (called the scattering force) pushes the bead along the direction of beam propagation. The second force the bead experiences emerges from the gradient force arising from the gradient of field intensity. 3D manipulation of the bead is possible by using these gradient and directional forces, which depend on the type of laser used. Specific details of the bead trapping mechanisms applied in biology are found elsewhere [127,128,129]. 

Forces produced by optical tweezers are within the range of 0.1 pico newton (pN) to ~100 pN [130], which makes it ideal for cellular and molecular level force probe applications. High-resolution optical traps are sensitive enough to measure nanometer scale displacement with micrometer size beads [131]. As a result, optical tweezers are an ideal tool for manipulating a bead attached to either DNA, proteins or enzymes. 

For whole cell studies, the beads were coated with a ligand of choice to specifically target receptors of interest on the cell surface. Once the bead is attached to a cell, the laser beam can be used to manipulate the bead attached to the cell surface. It is worth mentioning that the functionality of optical tweezers can be further improved by combining it with detection techniques such as confocal fluorescence, Förster resonance energy transfer (FRET), fluorescence lifetime imaging microscopy (FLIM), stimulated emission depletion (STED) or Raman [129].

### 2.4. Atomic Force Microscopy (AFM)

AFM (Atomic Force Microscopy, also known as scanning probe microscopy), like MTC, can be used to apply a controlled amount of force (pico to nano Newton level forces) on the cell surface. In AFM, a cantilever with a tip at one end is used to probe the cell. The displacement in the *z* direction of the cantilever is measured using a laser that reflects off the back surface of the cantilever beam. If the spring constant of the cantilever is softer than the surface being probed, the cantilever bends and the deflection is measured using a position sensitive detector. The cantilever beam bends when the tip shears or pulls on molecules that resist *z*-axis displacement or on completely extended cellular receptors, and in advanced systems, force-induced twist (angular twist) of the beam is also monitored. The calibrated cantilever often has a functionalized tip that carries a ligand (example RGD peptide to apply forces on integrin receptors on cells). By altering the ligand, forces can be applied specifically on certain cellular receptors. To probe biological samples, wet mode AFM is used where the kinetics of binding, unfolding and folding events can be monitored while the receptor is being sheared [20,132,133]. 

The three modes of AFM are contact mode, intermittent contact mode, and non-contact mode. In biological samples, probe tip extension and/or shear on bound cellular receptors produces surface shear in contact mode. The force-indentation curve (applied force vs. distance of the tip from the sample surface) is often fitted to a Hertz model of contact mechanics. Force curves reflect receptor properties of adhesion, elasticity, and bond rupture strengths [134]. The elasticity of the local tip-receptor interaction is calculated from the slope of beam deflection to tip displacement. The stiffness of the material (local volumetric region experiencing and resisting shear around the cell receptor) is measured as the ratio of applied force over strain along the direction of force. The forces are normalized to contact area to minimize the influence of material geometry on stiffness. For a perfectly elastic material, the linear relationship of strain and stress is given by Hooke’s law, *σ* = *E*·*ε*; where *σ* is the applied stress (*σ* = Force/contact area), *E* is Young’s modulus of elasticity, *ε* is produced strain (dl/l). In AFM compression experiments, using a spherical tip, when the applied force varies linearly with surface displacement, the applied force *P* is given by

$$P=\frac{4E}{3\left(1-\nu^{2}\right)}\sqrt{R}\;\delta^{3/2}$$

where *E* is elastic modulus, *R* is the radius of the indenter, *δ* is indentation depth and ν is the Poisson ratio for the material [135]. 

AFM can be modified to study single molecule experiments [20], where extension and conformation changes can be coupled with FRET pairs and the forces and strain within a molecule can be calibrated in tandem [18,124]. AFM has also been used to stretch biomolecules that involve intra-domain unfolding mechanisms with multiple conformations [133,136,137]. AFM has also been used to monitor the extent of modular trans/cis interaction and bond strength [138], association and dissociation rates [139], directionality of stretch [140], phosphorylation levels [141] and electrostatic potential at biological surfaces [142]. 

### 2.5. Shear Flow Microfluidic Devices

Parallel plate flow chamber experiments with endothelial cells have shown that shear flow can align cells [50,143,144,145,146,147,148]. To understand the influence of fluid shear, and to exert better control of the direction of shear and simultaneously monitor shear-induced changes efficiently, different microfluidic flow chambers have been designed. These devices apply a variety of mechanical stimuli like shear, stiffness gradients, confined zones, interstitial flow mimics and cell stretching [149,150,151,152,153]. For example, Song et al. developed a multi-compartment shear flow microfluidic platform using a braille piezoelectric array of pins that functions as valves with a multi-step peristaltic pumping sequence [149,154]. The channels control the shear stress by controlling the flow rate (volume displacement by the pump), which is indirectly controlled by the frequency of pumping sequence (braille pin movement). Using this setup, Song et al. [154] investigated the change in the angle of cell orientation and shape index (degree of roundness) by visualizing endothelial cell morphology (cell elongation and orientation). Another design mimicking interstitial forces between capillaries and tissues was built using a multi-channel microfluidic device that could effectively control hydrostatic pressures across an endothelial cell layer [149,151]. Cells were grown in 3D scaffold hydrogels assembled between two microchannels. The surface shear and interstitial forces (perpendicular to the cell plane) were controlled by regulating the cell growth media volume in the reservoir microchannels [151]. 

To quantify stretching (strain) effects on cells, microfluidic devices that can stretch cell substrates have been designed [152]. Here, a flexible and porous PDMS substrate sandwiched between two other PDMS layers adjoined by two vacuum chambers has been used to apply cyclic strain and stretch on bound cells (by regulating the magnitude and frequency of the vacuum and thus indirectly straining PDMS substrate). Another example is the physical application of uniaxial [155,156,157], biaxial [158] or equibiaxial [159,160] strain on cells by directly stretching their hydrogel supports. Confinement effects have also been studied using microfluidic channels that spatially control the 2.5D to 3D environment [153]. Confinement effects can directly assess the effect of confinement on cancer cell metastasis [150].

## 3. Adhesion Receptors that Transduce Force

The characteristic feature distinguishing multicellular organisms from unicellular ones is their ability to form functional multicellular aggregates—tissues with intercellular networks. Epithelial cells form 2D monolayers and can sense their external chemical and physical environment using specific membrane-associated receptors [4,15,34]. These receptors serve as bio-molecular antennas, receiving and transducing external chemical (ligand) and physical (mechanical, electrical) stimuli [8,161]. 

The hallmark of mechanotransduction is the cell’s biochemical adaptability to respond to external mechanical stimuli. This dynamic ability is regulated by the specificity of the receptor transducing the force [17,162,163,164], the activated mechano-biochemical pathway and the induced secondary messenger elements [11,18,27,56]. The kinetics of receptor-ligand binding [164,165,166], shear induced reactions [18,156,167,168] and pathways activated [23,143,168,169,170,171,172,173,174,175] are still being investigated using advanced biophysical tools coupled with imaging and biomolecular engineering techniques. 

Cells use adhesion receptors to sense substrates and neighboring cells. The dominant cell adhesion receptors include cadherins [176,177], integrins [172,178,179], immunoglobulin-like Cell Adhesion Molecules (ICAM) [179], and selectins [34]. The cadherin protein family comprises essential cell-cell adhesion proteins, and the integrin family forms cell-extracellular matrix adhesions. Here, we focus on the mechanotransduction pathways of cadherin and integrin receptors in epithelial cells.

### 3.1. Integrin—The Primary Cell-Matrix Adhesion Molecule 

Integrins enable cells to adhere and spread on extracellular matrices [172,180]. Each integrin receptor is a cis-heterodimer of α and β transmembrane proteins [172,181]. There is a total of 18 reported isoforms of α, and 8 isoforms of β receptors, and each specific α-β heterodimer can recognize one or more ECM protein [181]. The extracellular N-terminal region of the α receptor has an I domain (present in some α subunit isoforms), a seven-bladed β propeller domain, a thigh domain and the calf 1 and calf 2 domains. The extracellular N-terminal region of the β receptor has a β I like domain, a hybrid domain, cysteine rich plexin-semaphoring-integrin (PSI) domain, β sandwich domain, 4 epidermal growth factor (EGF)-like repeats and a β tail domain [172]. With the many isoforms of α and β receptors, there is a total of 24 known integrin α-β heterodimers that recognize specific extracellular matrix (ECM) molecules [182,183]. The dominant integrin recognized ECM proteins include fibronectin (RGD, LDV), collagen isoforms I and IV (GFOGER), laminin isoforms (YIGSR), vitronectin (RGD), fibrinogen (RGD), thrombospondin (RGD, NVR, LDVP), and other glycoproteins [184].

Among integrin ligands, the RGD peptide is the best characterized and most studied, with a third of functional integrin isoforms recognizing the RGD domain [181,185,186]. The RGD domain is found in multiple ECM proteins and is recognized by α5β1, αVβ3, αVβ1, αVβ5, αVβ6, αVβ8, and αIIbβ3 integrin heterodimers, laminin isoforms are recognized by α1β1, α2β1, α3β1, α6β1, α7β1 and α6β4, and collagen isoforms are recognized by α1β1, α2β1, α3β1, α10β1, and α11β1 [179,181,182]. Since integrins are capable of binding to multiple ECM peptides, it is the level of surface expression, relative ligand availability, affinity and trans-domination among the isoforms that determine the subtype of integrin-ECM adhesion [17,180,187]. Integrins have 2 major functional conformations that relate its affinity to ECM ligand: the inactive low affinity or bent conformation and the ligand bound high-affinity or extended conformation. Using FRET probes, a separation of 5 nm was observed between α and β stem domains (under Mn^2+^ stimulated active state) [188] while cryoelectron tomography studies did not detect any significant height changes (on integrin α_II_β_3_ activation [189]). The bent form (bent at an angle of 135°, between I-EGF-1 and EGF2 domain of β subunit) is inactive with its inability to bind to ligand (lower affinity structure) [190]. Exposure of receptor epitopes that interact with the ligand is higher in the upright conformation (higher affinity structure). Integrins, upon activation, provide an intracellular scaffold for the assembly of cytosolic signaling complexes. These intracellular signaling events that follow extracellular stimulation are commonly described as “*outside-in*” signaling events (compared to intracellular events that lead to integrin activation, called “*inside-out”* signaling) [190]. The biochemical events controlling integrin-cytoskeleton interactions have implications in many cellular processes like cell migration, membrane protrusion, proliferation, cancer metastasis, immune cell invasion, and apoptosis, among others. 

#### 3.1.1. Focal Adhesions

New integrin adhesions (nascent focal adhesions) that form from lamellipodial membrane extensions are very dynamic and contain talin and paxillin in the nascent complex [66]. Clustering of integrin receptors and the assembly of the adhesion complex enhance cell adhesion strength [173,191]. Talin serves as an actin tethering site, and the maturation of nascent adhesion complex forms focal complexes (FX) [192]. In addition to nascent adhesion complex proteins, FX includes a 100 nm diameter cluster complex that houses active vinculin, focal adhesion kinase (FAK), Src family kinases (SFK), α-actinin, actin related protein 2-actin related protein 3 complex (Arp2/3), and vasodilator-stimulated phosphoprotein (VASP) [192]. Mature focal adhesions (FA) contain active zyxin and tensin molecules in addition to focal complex components [192,193]. Initial nascent adhesions have low levels of vinculin and focal adhesion kinase with short actin interconnections. Vinculin activation and consequent binding to talin enables another connection between the integrin-ECM and the actin cytoskeleton [169,194]. As the actin fibers are reinforced with actomyosin-based contractility elements, the nascent adhesion matures and grows into a focal adhesion [66,195,196]. Maturation of focal adhesion is also associated with the accumulation of α-actinin in the complex that crosslinks actin filaments [197], and activation of RHO GEFs (LARG, GEF H1), which mediate myosin contraction through ROCK [162,198,199,200]. The literature highlighting the recruitment and activation of specific molecules at focal adhesions (which regulate cell morphology, signaling and behavior) is excellently tabulated in this article [201]. 

#### 3.1.2. Integrin’s Ability to Transduce Force

The role of cellular tension in modulating integrin function, integrin-actin linkage, and integrin-ECM adhesion has been extensively investigated [166,202]. The tension in and maturation of FX recruits talin and vinculin [192], which modulates the integrin mechanotransduction process through actin linkages [203]. The integrin mechanosensing process begins with cell adhesion and recognition of a substrate. Lamellipodial extensions occur via branched actin protrusions (force from actin polymerization pushes the membrane outward). Actin is linked to integrins through talin [204] and vinculin [124,205], and the myosin-generated actin tensile force is transmitted through integrins, which can indirectly induce conformation changes in ECM proteins [163]. The force range of integrin clusters within focal complexes was found to be above 54 pN using single molecule integrin tension sensors, while the tension in nascent complexes was ~40 pN [206]. 

Myosin-dependent traction forces modulate integrin’s role in sensing the substrate [207]. On soft ECM substrates, there is a lower density of actin stress fibers in cells, and reduced force across the ECM-integrin-cytoskeleton link [124,163]. The cortical actin filaments and integrin adhesions are nominally perpendicular to one other and force transmission from the actin cytoskeleton to the integrin complex is enabled by components that form a clutch [54]. Under high intracellular myosin-mediated contractility, actin moves backward (retrograde motion) [207] and transmits tensile force to integrins, which then pulls on the ECM (substrate). On harder substrates, the matrix resists the force from the cell and integrins remain relatively immobile, while on softer substrates the matrix is deformed [5,207]. FA complexes exhibit dynamic traction forces and the role of FAK, paxillin, and vinculin in the complex is essential for cell migration and rigidity sensing [207]. Integrins outside focal adhesions also exert traction forces, and using special multiplexed integrin tension gauge tethers, the exerted forces were found to be in the range of 43–54 pN, which was significantly lower than tensions within FA clusters [208]. 

Focal adhesions are associated with many signaling molecules on the cytoplasmic side [209]. Focal Adhesion Kinase (FAK) is a membrane-associated tyrosine kinase, and its activity is coupled with focal adhesion activity and maturation [173,201,210]. FAK is reported to regulate focal adhesion turnover, cell migration, crosstalk between growth factors and other integrin complexes [209,210,211]. FAK activation is a multistep process [212]—FAK is transiently activated (autophosphorylated at Y397) by integrin clustering and recruits SH2 binding Src kinase which phosphorylates Y576–577 and increases FAK activity 20-fold [209,210,213]. FAK is also reported to be biophysically activated by ECM, ligand density and substrate stiffness [163]. FAK can also phosphorylate and activate Src, and Src is reported to be active at integrin junctions [11,23,76,209]. Src has also been reported to be mechanically activated under integrin shear [92] and growth factor stimulation [214,215]. As to the Rho GTPases, Rac is associated with lamellipodial extension and actin protrusion at the membrane [216], while the Rho GEFs, namely LARG and GEF-H1, are activated indirectly by Rho kinase [162,198,199]. ROCK (Rho associated kinase) indirectly activates actomyosin-based contractility by inhibiting the phosphatase activity of MLCK phosphatase [199,217]. Cdc42 reportedly suppresses Rho GTPase activity and relieves intracellular tension, avoiding stress-induced detachment of cell-cell adhesions [11,218]. Rac and Rho activity are reciprocally correlated to contractility and actin dynamics in lamellipodial extension [219].

The temporal and spatial activity of the aforementioned FA-related kinases are currently being investigated in the context of mechanotransduction [23,92,162]. Since the complex network of kinases is spatially and temporally regulated in different activation states, the population average of active and inactive kinases needs to be controlled in respective assays. Normalization (±stimuli) of whole population averaged results may not be directly comparable since the relative change in kinase activity is time and location dependent. Different cell lines vary in the endogenous level of kinase activity and this may affect comparing results from cells of different tissue origin, mutations, and pathway modifications. Currently, calibratable kinase reporter assays with real-time quantification of results are used to decode the activation and functionality of kinases under specific stimuli [18,163,214,220,221]. 

### 3.2. Cadherin—The Primary Calcium Dependent Cell-Cell Adhesion Molecule 

Cell-cell adhesion is mediated by many adhesive receptors through lateral non-covalent interactions between neighboring cells [161]. The classical cadherin family of receptors dominates these lateral interactions, forming adherens junctions in epithelial cells. Tight junctions (claudin and occludin), gap and other desmosomal junction proteins are also present at lateral cell-cell contacts [18,177,222,223,224], but their role in mechanotransduction is not completely elucidated.

Cadherin has been well established as a cell-cell adhesion molecule, and with the advancement of biomechanical adhesion tools, new biomechanical functions of cadherin have been reported [100,225,226,227]. Cadherin is a single pass transmembrane protein, and the cadherin subfamily includes classical type 1, atypical type 2, desmosomal, protocadherins, Flamingo/Celsr, and Dachsous and Fat cadherins [35,177,228,229]. Here, we focus on the role of E-cadherin (a classical type 1 Cadherin) in mechanotransduction. E-cadherin has 5 extracellular domains (EC1–5), a transmembrane domain, and a cytosolic domain. The extracellular EC1–5 domains form active cis and trans adhesion complexes in the presence of calcium [177,230,231]. The juxtamembrane portion of cadherin interacts with p120 catenin [232], and the cytosolic domain (~76 amino acids) contains a direct binding site for β catenin [226,233,234], which in turn binds with α-catenin and allows the cadherin complex to indirectly associate with the actin microfilament [18,100,235,236]. The N-terminal end of α-catenin binds β-catenin, and the C-terminal of α-catenin binds with actin [100,235]. Microtubules are also reported to be associated with adherens junctions through the PLEKHA-NEZHA complex [237]. 

α-Catenin is a multi-helical repeat molecule. It has an autoinhibited M region between residues 376 to 633 [238] which is exposed to tension and myosin II activation [18,235,239]. The 906 amino acid long α-catenin shares functional and sequence homology with vinculin [240]. The major α-catenin domains are the N-terminal β-catenin binding domain ((57–146), a self-binding region (82–264)), M domain (376–633), and a C-terminal domain (697–906, binds to actin) [240,241]. There exists a serine-threonine responsive casein kinase (CK1, CK2) substrate within a phospho-linker region (M domain and C-terminal of α-catenin) that is reported to aid cell migration and wound closure [242]. Studies have shown that α-catenin can form a homodimer [227] and also has binding sites for actin and actin-binding molecules such as epithelial protein lost in neoplasm (EPLIN), vinculin, afadin, α-actinin, ZO-1, formin and zyxin [170,239,240,243]. The molecule can potentially recruit more actin fibers to cadherin junctions but requires actomyosin tension to unfurl and recruit vinculin [18,20,24,236]. The in vitro reconstitution of the actin*-*α-catenin-β-catenin-cadherin complex (without lateral actomyosin forces) revealed the biochemical inability of α-catenin to simultaneously interact with actin and β-catenin [227,244]. In vivo studies using time-resolved FRET and immunofluorescence [18,100], drosophila embryos experiments [239], and electron tomography experiments [236] showed that α-catenin can simultaneously interact with actin and β catenin at cadherin junctions under tension. This conundrum of opposing α-catenin in vivo and in vitro functions identified in the previous decade [227,233,243,244,245,246,247,248] was resolved recently using single molecule force probes and kinetic assays [245].

Vinculin is another molecule that aids cadherin adhesive activity. It is well known that vinculin shares sequence and structural homology with α-catenin [240], and binds to α catenin at the modulatory M domain [24,100,171,235]. Vinculin is directly recruited and activated in cadherin mechanotransduction [18,24,100], and is also studied as an actin linker protein at focal adhesions [205,249]. Its internal auto-inhibited domain is similar to that of α-catenin [168], which is activated through force and ligand-dependent activation [124]. Vinculin’s role at focal adhesion and cadherin junctions has raised the possibility that vinculin can regulate the crosstalk between adhesion receptors and global mechanotransduction [24,169,170]. Force dependent increase in actin and actin-associated protein recruitment, with improved cadherin junctional rigidity is known as the *cadherin mediated cell junctional stiffening response*. The multiple ways by which cadherin can associate with actin possibly showcases the ability of cells to control adherens junction rigidity and mechanosensitivity, depending on the context. 

Cell-cell adhesions resist intracellular contractile tension, protrusive forces from the membrane and extracellular forces on the cell. Like integrins, cadherins are also associated with actin (through catenins) and tension across adherens junctions is modulated by actomyosin-mediated contractility [50,250]. C- (Compaction stage), N- (neural), E- (epithelial) and VE- (vascular endothelial) cadherins exhibit local actin dependent junction remodeling and increased junction stiffness following cadherin-receptor perturbation [100,251]. The response of cadherin complexes to intracellular and external stress demonstrates that they are mechanosensors [252,253]. C2C12 myogenic cells (that have N-cadherin receptors) and Madin-Darby canine kidney (MDCK) epithelial cells (that have E-cadherin receptors) can specifically sense and respond to extracellular forces through respective N- and E-cadherin functionalized substrates or beads [24,251]. Cadherin mechanotransduction is also ligand-specific. α-catenin, vinculin, and actin get specifically activated and/or recruited in response to cadherin mechanotransduction [18,50,100].

The global time-resolved downstream molecular cascades regulating cadherin mechanotransduction are yet to be completely elucidated. In the model detailed by Yonemura et al. [235], α-catenin unfurls in response to increased tension on cadherin complexes, allowing actin engagement through vinculin recruitment [18,235]. In vitro experiments with E-cadherin and α-catenin FRET sensor expressing epithelial cells showed that α-catenin, vinculin, activated growth factor receptor, ligand specific interaction and actomyosin tension are required for cadherin-mediated stiffening response [18,24,236,254,255]. Cadherin activation with calcium, ligand-specific homophilic adhesion, cadherin cis or trans clustering, and conformation bond specific shear have been shown to activate downstream cadherin signals through Rho GTPases (Rac, Rho, Cdc42), Src, PI3K, FAK family of kinases [76,162,218,256,257,258], and respective protein tyrosine phosphatases [259,260,261]. The modulation of the spatial and temporal activity of these signaling molecules specifically due to cadherin mechanotransduction remains to be fully elucidated [199,218,257,261,262,263]. Cadherin malfunction and impaired mechanotransduction are involved in cardiomyopathies, vascular permeability deregulation, and lung injury among others [37,110,160,264,265,266]. 

### 3.3. Global Mechanotransduction

Mechanotransduction studies often rely on analyzing biochemical changes at the vicinity of the mechanical perturbation. Although the readout is measured at the site of applied forces, the specificity of the response and all the ensuing biochemical changes together define the complete mechanotransduction response. The term global mechanotransduction includes larger phenotypic cell-wide changes that occur away from the site of applied shear. Examples include a change in apical-basal cell polarity in response to fluid shear in endothelial cells [267], shear induced opening of calcium ion channels far from the site of shear [268], change in apical PECAM-1 stiffening response by altering basal integrin adhesions [162], creation of new basal focal adhesions by apical cadherin stimulation [255] and global RhoA activation under PECAM-1 shear [269]. The change in mechanical force balance between tensile (actin and intermediary filaments) and compressive (microtubule) elements in the cell (that resist and or respond to mechanical stresses [10,270]) are liable to induce global mechanical changes in the cell [271]. Collins et al. had discussed that PECAM-1 induced apical stiffening required new integrin adhesions basally [162], suggesting a biochemical regulatory phenomenon occurring away from the site of shear. These spatially and functionally isolated adhesive receptors (PECAM-1 and integrins) were biomechanically interacting to produce a PECAM dependent stiffening response. A similar mechanism was identified with E-cadherin as well [255]. 

Another example of global mechanotransduction is seen in murine kidney cells, where flow induced stresses activate calcium influx through the opening of ion channels [268]. Polycystin 1 is localized at the base of the primary cilium, which undergoes conformational changes under shear and opens up the calcium ion channel (Polycystin 2) [268]. The response is amplified by further release of intracellular calcium stores. The medium of force transduction between spatially isolated receptors was described in a *stress-focusing* model by Hu et al., using fluorescently labeled fiducial markers in the cytosol of Human airway smooth muscle cells [271]. The molecular events underlying the rapid mechanical crosstalk between spatially isolated receptors are still being investigated and will pave the way towards understanding tissue mechanotransduction.

### 3.4. Kinetic Regulation of Mechanotransduction

The phenomenon of mechanotransduction ensures a rapid biochemical response to external mechanical stimuli. In some mechanically stimulated pathways, the transduction of mechano-biochemical events is presumably faster than protein translocation and intracellular protein diffusion, in the order of milliseconds [92]. The biochemical cascade of events that follow mechanosensing is strictly regulated by multiple proteins that, for example, require recruitment, conformational changes, activation, (de-)phosphorylation, secondary messenger activation, binding to cytoskeletal structural elements, nuclear translocation, and/or gene expression. Each event is a controlled process that modulates the rate of the mechanotransduction response. The limiting factors regulating the cascade are often investigated using biochemical mutagenesis, siRNA, chemical inhibitors, single molecule biophysical, and kinetic studies. The kinetics can get complicated when the available concentration of each substrate, the conformation specific affinities and mixed parallel reactions of substrate/product and inhibitor kinetics have to be envisioned simultaneously in response to a specific mechanical stimulation. 

An example of a kinetic control element regulating cadherin mechanotransduction is α-catenin. The actin binding kinetics of α-catenin homodimer (α-α-catenin) vs heterodimer (β-α-catenin) was identified under biochemical reconstitution experiments in vitro [233,244]. α-catenin can either exclusively bind to β catenin or form α-catenin homodimers in vitro, as the α-β and α-α interacting domains have overlapping binding regions [227]. In solution, the affinity for α-catenin towards forming a homodimer is higher than α-catenin-actin interaction, and also competes with Arp2/3 branching of actin filaments [244]. Near the cell membrane, the local concentration of actin monomers at cadherin junctions is high, promoting α-catenin-actin interaction; and intracellular actomyosin-mediated contractile forces unfurl α-catenin and expose actin-binding domains. Tension appears to further increase α-catenin’s affinity to actin and also recruits vinculin with nanomolar affinity. The pseudo first order vinculin recruitment and binding to the cadherin adhesion complex were kinetically evaluated using AFM, mutagenesis, FRET, and stretch assays [18,20]. The aforementioned example illustrates that kinetic studies of mechanotransduction are more involved, but can reveal a dynamic and rich cellular response to mechanical stimulation.

## 4. Conclusions

This review is structured to enable students to quickly understand the basic experimental and conceptual fundamentals related to mechanotransduction studies. In this spirit, Table 1 further summarizes other reviews that can help introduce a student to relevant concepts and tools.

## Figures and Tables

**Table 1:** Summary of reviews.

Experimental Method/Aspect of Mechanotransduction	Representative References	Schematics/Tables That Explain Concepts/Theory
Mechanotransduction	[272,273]	Figure 1 from [9] and Figure 6 from [274]
Mechanotransduction diseases	[3]	Not applicable
Cytoskeletal mechanotransduction	[64,275]	Figure 4 from [203]
Traction Force/Stress Microscopy	[68,276]	Figure 4 from [63]
Magnetic Twisting Cytometry	[277]	Figure 1 from [277]
Microfluidic Shear	[149]	Figure 2 from [149]
Comparison of force application tools	[55]	Table 1 from [55,128]
Integrin mechanotransduction	[278]	Figure 4 from [5]
Cadherin mechanotransduction	[177]	Figure 1 from [9] & Figure 4 from [177]
Mechanotransduction signals	[28,34,176]	Figures 2–7 from [34]
